# Anesthetic management for withdrawal from a right ventricular assist device and Fontan procedure in a patient with an implantable left ventricular assist device for fulminant cardiomyopathy

**DOI:** 10.1186/s40981-023-00620-0

**Published:** 2023-05-26

**Authors:** Naka Kida, Masahiro Morinaga, Naoki Tadokoro, Takuma Maeda, Yoshihiko Onishi

**Affiliations:** 1 Department of Anesthesiology, National Cerebral and Cardiovascular Center, 6-1 Kishibeshinmachi, Suita City, 564-8565 Osaka, Japan; 2 Department of Cardiovascular Surgery, National Cerebral and Cardiovascular Center, Osaka, Japan

**Keywords:** Right ventricular assist device withdrawal, Implantable left ventricular assist device, Fontan procedure, Inflow cannula, Suck-down event

## Abstract

**Background:**

We herein report the anesthetic management for extracardiac conduit–total cavopulmonary connection (EC-TCPC) for weaning from an extracorporeal right ventricular assist device (RVAD) in a patient with an implantable left ventricular assist device (LVAD) for fulminant cardiomyopathy.

**Case presentation:**

A 24-year-old man developed fulminant cardiomyopathy and was placed on a biventricular assist device (BiVAD) comprising an implantable LVAD and an extracorporeal RVAD. The Fontan procedure was performed to wean the patient from the RVAD and allow him to be discharged home. Atrial septal defect creation, right ventricular suture, and tricuspid valve closure were then simultaneously performed to ensure sufficient left ventricular preload to drive the LVAD. Furthermore, to keep the central venous pressure lower, the inflow cannula of the LVAD was oriented in the correct direction.

**Conclusion:**

This is the first report of anesthetic management of the Fontan procedure in a patient with a BiVAD.

**Supplementary Information:**

The online version contains supplementary material available at 10.1186/s40981-023-00620-0.

## Background

The waiting period for a heart transplant in Japan is almost 3.8 years, longer than that in Western countries because of the small number of heart transplantation donors. Patients are able to be discharged home with implantable left ventricular assist devices (LVADs) such as the HeartMate 3 (HM3) (Abbott, Abbott Park, IL, USA) until heart transplantation is performed. However, patients with right ventricular assist devices (RVADs) are forced to remain in the hospital before heart transplantation because implantable RVADs have not been approved in Japan, in contrast to Australia [1]. Implantation of an LVAD and extracardiac conduit–total cavopulmonary connection (EC-TCPC) were recently performed in patients with biventricular assist devices (BiVADs) to facilitate weaning from an RVAD [2]. The most important consideration when introducing a Fontan circulation to a patient with an HM3 is to ensure sufficient left ventricular preload to drive the HM3.

This is the first report of the anesthetic management of EC-TCPC, atrial septal defect (ASD) creation, tricuspid valve resection, and right ventricle suture in a patient with a BiVAD.

## Case presentation

A 24-year-old man was scheduled for EC-TCPC, tricuspid valve resection, right ventricle suture, ASD creation, and RVAD weaning for fulminant cardiomyopathy with a BiVAD (HM3 and extracorporeal RVAD).

**Fig. 1:**
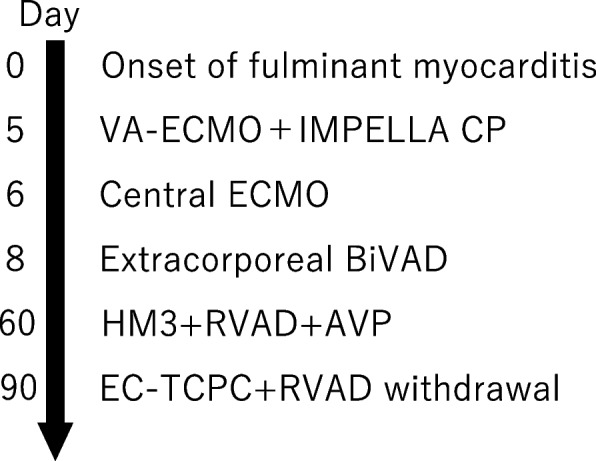
Clinical progression until surgery. The patient had no medical history. He developed malaise on day 0 and fever on day 1. On day 4, the patient was diagnosed with fulminant cardiomyopathy by the previous physician and administered catecholamines. On day 5, he was treated with VA-ECMO and an Impella CP left ventricular assist device (Abiomed Inc., Danvers, MA, USA). On day 6, the patient was transferred to our hospital for more advanced treatment. ECMO, extracorporeal membrane oxygenation; VA-ECMO, blood is extracted from the right atrium and returned to the arterial system, bypassing the heart and lungs; Central ECMO, blood is extracted from the right atrium and left ventricle and returned to the pulmonary artery and aortic artery, bypassing the heart and lungs; BiVAD, biventricular assist device; HM3, HeartMate 3 implantable left ventricular assist device (Abbott); RVAD, right ventricular assist device; AVP, aortic valve plasty; EC-TCPC, extracardiac conduit–total cavopulmonary connection

Figure 1 shows the patient’s clinical progression up to the surgery. Preoperative electrocardiography showed ventricular tachycardia with a heart rate of 300 beats/min. Preoperative echocardiography showed a left ventricular dimension in diastole of 40 mm, severely reduced left and right ventricular contraction, and trivial aortic valve regurgitation after aortic valve plasty. Supplemental Table 1 shows the RVAD weaning test results. Although the cardiac index was maintained after withdrawal of the RVAD, EC-TCPC surgery was scheduled to prevent the left ventricular narrowing and sucking-down events caused by right ventricular enlargement due to the HM3. In addition, there was concern that introducing a Fontan circulation into the biventricular space would cause right ventricular enlargement due to blood flow from the coronary sinus and small cardiac veins if only pulmonary artery blockade and ASD creation were performed. Therefore, we decided to perform right heart stenosis and tricuspid valve closure.

**Fig. 2:**
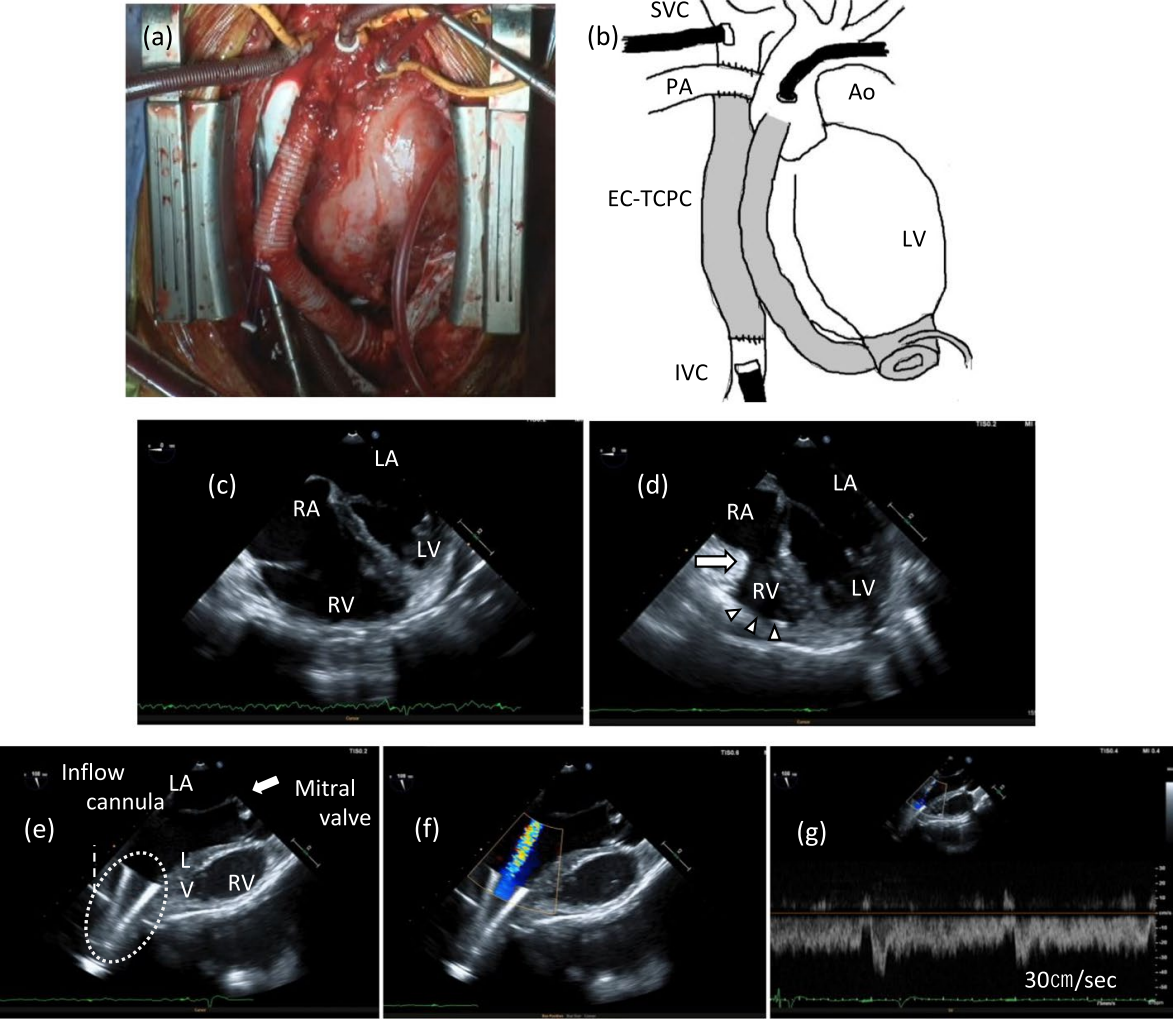
Surgical field and TEE findings. **a** Findings in the operative field before withdrawal of cardiopulmonary bypass. **b** Schema of the operative field before withdrawal of cardiopulmonary bypass. **c** Preoperative TEE findings. **d** TEE findings before weaning from cardiopulmonary bypass. ( →) after tricuspid valve closure, (△) after right ventricle suture. **e** Inflow cannula is oriented toward the mitral valve. **f** Inflow cannula shows no stenosis in color flow Doppler. **g** No acceleration of blood flow in continuous wave Doppler. TEE, transesophageal echocardiography; SVC, superior vena cava; PA, pulmonary artery; Ao, aorta; EC-TCPC, extracardiac conduit–total cavopulmonary connection; RV, right ventricle; LV, left ventricle; IVC, inferior vena cava

Cardiopulmonary bypass (CPB) was established, a cardioplegic solution was injected simultaneously with aortic cross-clamping, and a right atrial incision was made. The right ventricle was sutured from the inside, and tricuspid valve resection was performed. ASD creation was performed, and the right atrium was closed. The superior vena cava (SVC) and inferior vena cava (IVC) with an epicardial conduit were sutured to the pulmonary artery. Figure 2 shows the operative field findings before weaning from CPB.

Before weaning from CPB, we noted bilateral pleural effusions, which were aspirated by the surgeon; we also administered 20 ppm of nitric oxide (NO) to the patient to lower the pulmonary vascular resistance (PVR) and subsequently the central venous pressure (CVP). However, we administered neither catecholamines nor phosphodiesterase III inhibitors. After weaning from CPB, we confirmed by transesophageal echocardiography (TEE) that the ventricular septum was not deviated to the left and was not compressing the left ventricle due to the right heart suture (Fig. 2). We also confirmed by TEE that the inflow cannula of the HM3 had no obvious positional abnormalities and no accelerated blood flow (Fig. 2). There was no obvious stenosis at the anastomosis between the SVC, IVC, and pulmonary artery. The patient stopped receiving NO and was extubated 5 h postoperatively, and he was discharged from the intensive care unit 5 days postoperatively.

Table 1 shows the patient’s perioperative parameters and vital signs. At 2 months postoperatively, the patient was able to walk in the hospital room.

**Table 1:** Perioperative pressures

	Before surgery (ventilation)	After CPB (ventilation)	After surgery (ventilation)	POD6 (spontaneous)
HR (bpm)	320 (VT)	0 (asystole)	0 (asystole)	0 (asystole)
RVAD speed (rpm)	3010	-	-	-
RVAD flow (L/min)	3.5	-	-	-
HM3 speed (rpm)	5400	5400	5800	5800
HM3 flow (L/min)	3.8	4	4.6	4.8
mABP (mmHg)	62	62	63	53*
mLAP (mmHg)	-	5	-	7*
mRAP (mmHg)	15	14	15	13
ScvO_2_ (%)	62	72	68	74*

*HR* Heart rate, *RVAD* Right ventricular assist device, *HM3* HeartMate 3, *mABP* mean arterial blood pressure, *mLAP* mean left atrial pressure, *mRAP* mean right atrial pressure, *ScvO*_*2*_ Central venous oxygen saturation, *CPB* Cardiopulmonary bypass, *VT* Ventricular tachycardia, *POD* Postoperative day

^*^ABP was substituted for noninvasive blood pressure, LAP for mean pulmonary artery wedge pressure, and ScvO_2_ for mixed venous oxygen saturation

## Discussion

In this case, the EC-TCPC procedure was chosen because of concerns about right ventricular enlargement, unbalanced shifting of the ventricular septum, and an inadequate left ventricular volume following RVAD withdrawal. It is important to maintain the left chamber capacity to ensure adequate preload for driving the HM3 and thus prevent a suck-down event of the inflow cannula in patients with a Fontan circulation. Pulmonary artery dissection and ASD creation alone increase the risk of an inadequate left ventricular volume and occurrence of a suck-down event due to right ventricular enlargement caused by blood flowing from the coronary sinus and small cardiac veins. To decrease this risk, our patient underwent EC-TCPC and ASD creation as well as right ventricle suture and tricuspid valve resection.

To maintain adequate left ventricular volume, one of two patients in a previous report underwent simultaneous ventricular septal defect (VSD) creation with placement of an implantable LVAD and the other underwent plication of the right atrium and ventricle, as in this case [2]. In general, VSD creation may result in damage to the atrioventricular valve or conduction tract [3]. Additionally, VSD creation under cardiac arrest is different from that under a beating heart, and the VSD diameter may be smaller than expected. In this case, because of the ventricular tachycardia, there was no need to consider conduction defects; however, mitral valve damage and limitation of the VSD diameter were considered. Therefore, right heart suture and tricuspid valve closure were performed instead of VSD creation.

In the Fontan circulation, the probability of liver disease increases if the patient’s CVP remains above 15 mmHg, and mortality increases if the CVP remains above 20 mmHg [4]. Therefore, it is necessary to manage patients with an HM3 to maintain a low atrial pressure and low transpulmonary gradient (TPG) in order to keep the CVP low. First, to maintain low atrial pressure, we must confirm the abnormal position of the inflow cannula by TEE [5]. Inflow cannula malposition should be suspected when the following occurs despite an increased HM3 rpm: no increase in pump flow, suck-down events, sporadic premature ventricular contractions, and inability to maintain systemic blood pressure. In this case, the inflow cannula was in an appropriate position on TEE, pointing vertically from the apex toward the mitral valve (Fig. 2) and facilitating adequate unloading of the left intraventricular volume.

Second, our patient underwent aspiration of bilateral pleural effusions, and NO was administered prior to CPB withdrawal to manage a low TPG. A low TPG also suggests low PVR, which preserves blood flow to the left ventricle. Anesthesiologists can adjust several factors to lower the PVR: 100% fraction of inspired oxygen, positive end-expiratory pressure sufficient to maintain a functional residual air capacity [6] and minimal tidal volume, correction of respiratory and metabolic acidosis, optimization of the hemoglobin concentration [4, 7], and administration of phosphodiesterase III inhibitors and/or NO.

What differentiates this case from a simple LVAD or simple Fontan procedure is that we did not use inotropes and managed the circulation by controlling the intravascular volume, PVR, and systemic vascular resistance. We managed anesthesia by checking the central venous oxygen saturation and lactate concentration to identify circulatory failure. Tables 2 and 3 summarize the anesthetic management of the HM3 and Fontan circulation.

**Table 2:** Fontan circulation management

・TEE	Single ventricle heart function
	Atrioventricular valve regurgitation
	Stenosis of SVC/IVC anastomosis
	Pulmonary artery stenosis
・CVP	Target < 15 mmHg (< 20 mmHg just after surgery)
	Fenestration consideration if CVP is high
	Varies with factors of infusion volume, atrial pressure, and TPG
・Atrial pressure	Target < 10 mmHg (< 15 mmHg just after surgery)
	Infusion volume and use of inotropic drugs while checking Lactate and SvO2
・TPG	Target < 10 mmHg
	Prevention of atelectasis
	Appropriate PEEP or single ventilation volume
	Normocapnia

*TEE* transesophageal echocardiography, *SVC* superior vena cava, *IVC* inferior vena cava, *CVP* central venous pressure, *TPG* transpulmonary gradient, *SvO*_*2*_ mixed venous oxygen saturation, *PEEP* peak end-expiratory pressure

**Table 3:** LVAD management (continuous flow, centrifugal pump)

・TEE	Orientation of inflow cannula: perpendicular to mitral valve
	PFO: Draws blood flow from the right ventricular system into the lef
	t ventricular system after VAD drive
	MR: Left ventricular chamber is smaller after VAD drive, MR may be
	AR: Aortic valvuloplasty recommended if more than mild (Feldman)
	AS: Valve replacement recommended regardless of the stage
	TR: Tricuspid valve plasty is recommended in cases of moderate or severe valve ring enlargement
	MS: Less frequent, but if present, mitral valvuloplasty is recommended
	Air in left ventricular: prevent air emboli
	Ventricular septal deviation (see below)
・Blood pressure	Target: 65–75 mmHg
	< 60 mmHg: coronary return pressure decreases and right heart function declines
	> 80 mmHg: greater lift and reduced VAD flow
・Ventricular septum shift	Right shift (possibility of pulmonary congestion)
	Increase VAD rpm, check for AR and inflow cannula, blood pressure optimization
	Leftward shift (possibility of sucking down event)
	Decrease VAD rpm, check for inflow cannula, blood pressure optimization
	Correction of hypovolemia, use of inotropic drugs or NO

*LVAD* Left ventricular assist device, *TEE* Transesophageal echocardiography, *SVC* Superior vena cava, *IVC* Inferior vena cava, *PFO* Patent foramen ovale, *MR* Mitral regurgitation, *AR* Aortic regurgitation, *AS* Aortic stenosis, *TR* Tricuspid regurgitation, *MS* Mitral stenosis, *VAD* Ventricular assist device, *rpm* revolutions per minute, *NO* Nitric oxide

Although perioperative management and the long-term results of patients undergoing Fontan surgery with LVADs, such as this case, are unknown, many procedures for LVAD implantation in patients with a Fontan circulation have been reported. The postoperative management of these patients was similar to that of patients with a Fontan circulation [4]. It is recommended to monitor the atrial pressure and CVP and to keep the CVP below 15 mmHg. In particular, to lower the TPG, early extubation is recommended in addition to the above-mentioned medications and ventilator settings [8].

Cedars et al. [9] reported that 1 year after VAD implantation in patients with a Fontan circulation, 69.5% of patients had undergone transplantation and that 9.2% continued to be supported, whereas 21.3% of patients died while under VAD support. At least one postoperative complication occurred in 69% of patients, with the most common complications being neurologic dysfunction (25%), hemorrhage (23%), and infection (22%) [9].

The clinical criteria for performing the Fontan procedure in patients with LVADs are not clear. For patients who have fulminant cardiomyopathy with an initially low PVR, we consider it highly likely that the Fontan circulation and LVAD circulation will be viable. However, whether the Fontan circulation and LVAD circulation will be viable in patients with chronic left heart failure complicated by pulmonary hypertension is difficult to determine.

This surgery to convert patients with biventricular heart failure to an implantable LVAD and Fontan circulation may be one way to facilitate discharge home while waiting for heart transplantation in Japan.

## Supplementary Information

**Additional file 1: Supplemental table 1. **RVAD Weaning Test

## Abbreviations

LVAD: Left ventricular assist device
HM3: HeartMate 3
RVAD: Right ventricular assist device
EC-TCPC: Extracardiac conduit–total cavopulmonary connection
BiVAD: Biventricular assist device
ASD: Atrial septal defect
CPB: Cardiopulmonary bypass
SVC: Superior vena cava
IVC: Inferior vena cava
NO: Nitric oxide
PVR: Pulmonary vascular resistance
CVP: Central venous pressure
TEE: Transesophageal echocardiography
VSD: Ventricular septal defect
TPG: Transpulmonary gradient
