# Supplementary material for: Anesthetic management for withdrawal from a right ventricular assist device and Fontan procedure in a patient with an implantable left ventricular assist device for fulminant cardiomyopathy
Source: JA Clin Rep. 2023 May 26;9:31. doi: 10.1186/s40981-023-00620-0 (PMC10219899; doi:10.1186/s40981-023-00620-0)
Supplement: Supplementary file 1 — Additional file 1: Supplemental table 1. RVAD Weaning Test [file 40981_2023_620_MOESM1_ESM.docx]

**Supplemental Table 1. RVAD Weaning Test**

| HR (bpm) | 320(VT) | 320(VT) |
| --- | --- | --- |
| HM3 speed (rpm) | 5400 | 5800 |
| HM3 Flow (L/min) | 3.9 | 4.6 |
| ｍNIBP (mmHg) | 80 | 92 |
| ｍPAWP (mmHg) | 10 | 6 |
| ｍPAP (mmHg) | 15 | 12 |
| ｍRAP (mmHg) | 15 | 13 |
| SVO_2_(％) | 50.6 | 56.4 |
| PVR(Wood Unit) | 1.4 | 1.4 |
| CO (L/min) | 3.58 | 4.25 |
| CI (L/min/m^3^) | 2.15 | 2.54 |

RVAD: right ventricular assist device, VT: ventricular tachycardia, HR: heart rate, HM3: HeartMate 3, mNIBP: mean noninvasive blood pressure, mPAWP: mean pulmonary artery wedge pressure, mPAP: mean pulmonary artery pressure, mRAP: mean right atrial pressure, SvO_2_: mixed venous oxygen saturation, PVR: pulmonary vascular resistance (Wood units: mmHg/min/L), CO: cardiac output, CI: cardiac index
